# Psychosocial Resources and Emotional Support Needs in Women with Vulvodynia: A Lifespan Developmental and Biopsychosocial Perspective

**DOI:** 10.3390/bs15121600

**Published:** 2025-11-21

**Authors:** Valentina Lucia La Rosa, Elena Commodari

**Affiliations:** Department of Educational Sciences, University of Catania, 95124 Catania, Italy; elena.commodari@unict.it

**Keywords:** development, lifespan, women’s health, well-being, vulvodynia

## Abstract

Vulvodynia is a chronic vulvar pain condition that can interfere with women’s developmental processes and overall well-being. Adopting a broader perspective of women’s health informed by lifespan developmental and biopsychosocial frameworks, this study examined psychosocial factors related to the psychological well-being of Italian women with vulvodynia. Between December 2023 and December 2024, a total of 533 women diagnosed with vulvodynia completed an online survey. The survey included questions about sociodemographics and the illness, as well as validated measures of dyadic adjustment, social support, self-efficacy, perceived stress, and psychological well-being. Descriptive statistics, group comparisons, Pearson correlations, and hierarchical multiple regressions were performed. Nearly two-thirds of the women reported symptoms lasting over five years, and 44% experienced severe pain. Those with more intense pain, longer symptom duration, or delayed diagnosis reported lower well-being and higher stress. Satisfaction with treatment was linked to greater well-being. Psychological well-being was strongly correlated with social support, dyadic adjustment, and psychological resources. Regression analyses identified younger age, higher pain intensity, lower treatment satisfaction, reduced social support, lower self-efficacy, and greater stress as predictors of poorer psychological well-being. Vulvodynia should be considered a psychosocial and developmental challenge as well as a medical condition. These findings underscore the importance of viewing vulvodynia as not only a medical condition, but also a psychosocial and developmental challenge within women’s broader health trajectories, highlighting the need for interventions that address pain and provide structured emotional support to strengthen psychological and relational resources.

## 1. Introduction

Women’s health and well-being are multidimensional concepts that change across the lifespan, influenced by the continuous interaction of biological, psychological, and social factors. Health is not simply the absence of disease; rather, it is the ability to pursue meaningful goals, maintain fulfilling relationships, and exercise autonomy in ways that are consistent with developmental tasks and cultural expectations (Marmot, 2015; World Health Organization, 2020). This broader perspective of health is particularly relevant when considering women’s experiences, as they navigate both universal developmental transitions and gender-specific challenges that can significantly impact well-being (Chrisler, 2012; Ussher, 2006).

Throughout life, women experience age-specific transitions that are essential to identity development and social integration. During adolescence and emerging adulthood, individuals often focus on exploring their identity, developing autonomy, and establishing intimate relationships (Arnett, 2000). In early and mid-adulthood, consolidating romantic partnerships, advancing in career, and, in many cases, becoming a parent become central life tasks (Schulenberg et al., 2004b). Later stages bring new challenges, such as adapting to biological changes, negotiating intergenerational roles, and preserving autonomy in the context of aging (Baltes et al., 2006). At each stage, women’s well-being depends on their ability to successfully negotiate these tasks, which in turn requires both personal resources and supportive social contexts (Keyes, 2002).

Nevertheless, well-being throughout a woman’s life is not solely determined by normative developmental transitions. Women also face gender-based health disparities that arise from biological factors, societal expectations, and systemic inequalities in healthcare. For example, women are disproportionately affected by chronic pain, autoimmune disorders, and mental health issues, but their symptoms are frequently overlooked or dismissed in medical settings (Samulowitz et al., 2018; World Health Organization, 2020). Furthermore, cultural norms surrounding femininity, caregiving, and sexuality also influence how women experience, disclose, and manage health conditions. These inequalities highlight the importance of examining women’s health within an integrated framework that considers illness and adaptation within developmental and sociocultural contexts.

In this perspective, psychological and social resources play a central role in sustaining women’s well-being across the life course. Among these, self-efficacy, or beliefs about one’s ability to organize and carry out actions, supports the selection, initiation, and maintenance of effective responses to everyday demands (Bandura, 1997). Coping refers to the cognitive and behavioral efforts mobilized to manage these demands (Lazarus & Folkman, 1984). Stress management is a specific subset of coping strategies that is typically emotion-focused (Lazarus, 1993b). Social support, on the other hand, refers to the structural and functional resources provided by close others and communities (Uchino et al., 1996). It is not a coping response itself, but rather a contextual scaffold that buffers stress and amplifies the effectiveness of coping strategies over time (Lazarus, 1993a). When these resources are lacking, women are at greater risk of developmental stagnation, heightened distress, and a reduced sense of agency (Hendry & Kloep, 2002). It is important to underline that these processes are dynamic; resources can be mobilized, depleted, or transformed over time, depending on the nature of the challenges encountered and the quality of surrounding environments.

Two theoretical perspectives offer valuable insights into how women’s health and well-being evolve throughout their lives. The Lifespan Developmental Model (Hendry & Kloep, 2002) conceptualizes development as a lifelong negotiation of tasks and challenges with available resources. Within this framework, health conditions are considered as non-normative challenges: unexpected events that disrupt typical developmental pathways such as forming stable partnerships, advancing in one’s career, or becoming a parent. For example, the onset of a chronic illness in early adulthood may delay or derail identity exploration and the establishment of intimacy, while illness in midlife may interfere with caregiving responsibilities and work–life balance. When personal and contextual resources are insufficient, such challenges can lead to developmental stagnation; when resources are adequate, they may promote resilience and adaptive growth (Masten & Wright, 2010).

The biopsychosocial model (Engel, 1977) complements this developmental perspective by emphasizing the integration of biological, psychological, and social processes in influencing health outcomes. This model has been widely applied to chronic illness, emphasizing that symptoms and outcomes cannot be explained by biomedical factors alone (Wade & Halligan, 2017). Applied to women’s health, this model highlights that well-being emerges from the interplay of symptom severity, coping strategies, relational resources, and healthcare experiences, all of which are situated within specific stages of development. Together, these frameworks offer a powerful perspective on women’s adaptation to health-related challenges. In fact, they emphasize that well-being depends on more than just medical management, but also on psychological resources, relational support, and the capacity to integrate illness into broader life trajectories. Furthermore, they stress that the developmental timing of illness onset is crucial, since challenges that occur during sensitive life stages can have enduring consequences for well-being and social participation (Helgeson & Zajdel, 2017).

A paradigmatic example of a non-normative health challenge in women’s lifespan is *vulvodynia*. Defined as chronic vulvar pain persisting for at least three months without an identifiable cause, vulvodynia is a condition that is often misunderstood and underdiagnosed (Bornstein et al., 2016; Haefner et al., 2005). Prevalence estimates suggest that between 8% and 28% of women of reproductive age experience symptoms consistent with vulvodynia (Amirthalingam & Sivalingam, 2024; Harlow et al., 2014). In Italy, recent estimates suggest a prevalence of 12–15%, though this figure is likely conservative due to the stigma surrounding the condition, the limited awareness among healthcare professionals, and the absence of standardized diagnostic pathways (Gattamelata et al., 2025; Lountzi et al., 2025a). Vulvodynia has a multifactorial etiology involving peripheral and central sensitization, inflammatory and immunological mechanisms, pelvic floor hyperactivity, hormonal influences, genetic predispositions, and psychosocial factors such as stress or adverse sexual experiences (Lountzi et al., 2025a; C. F. Pukall et al., 2016). Yet the impact of the condition extends far beyond physical pain. Women often report disruptions in intimacy, reduced sexual satisfaction, and challenges to their sense of femininity and identity (Montali et al., 2025; Shallcross et al., 2017). Over time, vulvodynia can erode self-efficacy, foster social withdrawal, and constrain life projects, thereby undermining women’s ability to meet normative developmental tasks. Notably, vulvodynia frequently emerges during early or mid-adulthood, a period when women are typically establishing romantic relationships, pursuing educational and occupational goals, and often considering motherhood (Arnett, 2000). Thus, vulvodynia represents not only a medical concern but also a developmental challenge that intersects with key life transitions. Long diagnostic delays and insufficient healthcare responses exacerbate this burden, contributing to uncertainty, stigma, and prolonged distress (Shallcross et al., 2017).

Despite its profound implications, research on vulvodynia has largely focused on biomedical and epidemiological perspectives. Few studies have used developmental frameworks to examine how women adapt their goals, relationships, and sense of agency when dealing with a chronic and frequently misperceived painful condition (Montali et al., 2025). Recent contributions have expanded our understanding of the psychosocial and relational dimensions of vulvodynia. Systematic evidence indicates that psychological factors, including emotional distress, pain-related cognitions, and interpersonal dynamics, play a central role in shaping women’s experiences of pain and sexual functioning (Chisari et al., 2021). Qualitative studies further demonstrate that help-seeking trajectories are significantly impacted by partner responses and the quality of interactions with healthcare professionals, especially when symptoms are not widely recognized or dismissed (Lountzi & Durand, 2024). These findings align with early intervention research demonstrating that brief psychosocial or educational support can alleviate the emotional distress associated with vulvar pain and enhance women’s comprehension of their symptoms (Moravek et al., 2023). Complementary evidence also confirms the benefits of multidisciplinary therapeutic approaches that address the physical and psychological aspects of vulvodynia (Penteado et al., 2024). At the same time, the field is increasingly acknowledging the need to broaden its research agenda. A recent Delphi study identified psychosocial mechanisms, relational processes, and long-term patterns of adaptation as key priorities for advancing vulvodynia research in the coming years (Lountzi et al., 2025b), aligning with a growing body of work pointing to broader psychosocial barriers women commonly encounter, including stigma, relationship strain, and fragmented care pathways, which may hinder timely diagnosis and effective coping (Niedenfuehr et al., 2023). Together, these emerging contributions underscore the importance of examining vulvodynia through a multidimensional, developmentally informed lens that captures how women navigate a chronic pain condition within the contexts of their relationships, social environments, and broader life trajectories.

However, despite the increasing recognition of the psychosocial aspects of vulvodynia, factors such as social support, relationship quality, and psychological resources have rarely been examined systematically throughout the lifespan from a biopsychosocial perspective. This study aims to address this gap by investigating the psychosocial correlates of well-being in Italian women with vulvodynia. Using the Lifespan Developmental Model and a biopsychosocial framework, we examined how characteristics related to the illness, psychological resources, and social contexts intersect to shape women’s well-being. Based on these theoretical premises, we developed a primary hypothesis about the relationship between psychosocial factors and psychological well-being in women with vulvodynia. Specifically, we expected stronger psychosocial resources, such as higher perceived social support, greater dyadic adjustment, higher self-efficacy, and lower perceived stress, to be associated with better psychological well-being. Conversely, we anticipated that factors related to the illness, such as higher pain intensity, longer symptom duration, and prolonged diagnostic delays, would be linked to lower well-being. Our hypothesis reflects a lifespan and biopsychosocial perspective, suggesting that women’s adjustment to vulvodynia is shaped by the interaction between their personal resources, their relationships, and the challenges of living with a chronic pain condition.

## 2. Materials and Methods

### 2.1. Transparency and Openness

We adhered to the Transparency and Openness Promotion (TOP) guidelines (Nosek et al., 2015) when conducting and reporting this study. The study was not preregistered on a public registry (e.g., OSF Registries). Data collection was conducted between December 2023 and December 2024. All measures, variables, and analytic procedures are fully disclosed in the manuscript. Due to privacy constraints, the full dataset cannot be made publicly available. However, the corresponding author can provide anonymized data that support the findings of this study upon reasonable request. All statistical analyses were conducted using IBM SPSS Statistics, version 31.0 (IBM Corporation, Armonk, NY, USA).

### 2.2. Participants

A total of 533 Italian women participated in the study. Women were considered eligible if they met the following criteria: (1) age of at least 18 years; (2) a certified medical diagnosis of vulvodynia, (3) adequate proficiency in the Italian language, and (4) provision of valid informed consent. No further exclusion criteria were specified.

The mean age of participants was 34.6 years (SD = 10.9), with ages ranging from 18 to 64. Women were distributed across all Italian regions, with the largest proportions residing in Lombardy (16.9%), Lazio (12.6%), Veneto (9.9%), and Sicily (9.8%). The majority identified as heterosexual (92.1%), while smaller proportions identified as bisexual (6.6%) or homosexual (1.3%). In terms of relational status, 81.6% reported being in a romantic relationship, most of whom were either cohabiting (28.3%) or married (26.3%). Educational attainment was diverse, with 37.7% holding a high school diploma, 22.7% a bachelor’s degree, and 24.4% a master’s degree. Occupationally, the largest groups were employees (33.2%), followed by students (16.7%), professionals (11.8%), and academic or school staff (10.7%).

### 2.3. Procedure

The survey was administered online through Google Forms between December 2023 and December 2024, to ensure the recruitment of a large and diverse sample and strengthen the robustness of the findings. All instruments were administered in Italian. Whenever available, validated Italian versions were used. Items developed specifically for this study were created directly in Italian and reviewed by experts to ensure linguistic accuracy and cultural appropriateness for Italian women living with vulvodynia.

Participants were recruited through online platforms, including social media groups dedicated to vulvodynia, as well as through national and regional support associations. Participation was entirely voluntary, with no form of compensation, and anonymity and confidentiality were carefully maintained throughout the process.

Before accessing the questionnaire, participants provided electronic informed consent through a dedicated online form. To further safeguard data quality, the survey platform was configured to block multiple submissions from the same IP address. and the dataset was subsequently subjected to a careful manual review to identify and remove incongruent or duplicate responses based on demographic characteristics and response patterns. On average, completion of the survey required approximately 20 to 25 min.

The study was conducted in accordance with the ethical principles of the Declaration of Helsinki, the Code of Ethics of Italian Psychologists (Law No. 56, 18 February 1989), the Italian Data Protection Law (Legislative Decree 196/2003), and the Code of Ethics for Psychological Research (27 March 2015), as endorsed by the Italian Association of Psychology. The Internal Ethics Review Board of the Department of Educational Sciences at the University of Catania reviewed and approved the study protocol (approval code: n° Ierb-Edunict-2023.05.23/04).

### 2.4. Measures

#### 2.4.1. Sociodemographic and Illness-Related Variables

Participants provided sociodemographic information, including age, region of residence, educational attainment, occupational status, relationship status, marital status, and sexual orientation. To further contextualize their health experience, information on a range of illness-related variables was also collected. These included the duration of symptoms, the time elapsed between symptom onset and the formal diagnosis of vulvodynia, the types of symptoms reported, and the presence of comorbid medical or psychological conditions. The intensity of vulvar pain at the time of the survey was assessed using a Numerical Rating Scale (NRS) ranging from 0 (*no pain*) to 10 (*worst imaginable pain*). The NRS is among the most widely used instruments for assessing pain intensity across medical conditions, including vulvodynia (C. Pukall et al., 2025). Finally, participants were asked about their current therapeutic status and, if applicable, to indicate the type of treatment they were undergoing, such as pharmacological interventions, physiotherapy, psychotherapy, or multimodal approaches.

To further explore women’s experiences of care, two additional dimensions were considered: satisfaction with healthcare professionals and satisfaction with treatments. These measures were adapted from the corresponding sections of the *Endometriosis Health Profile-30* (EHP-30) (Jones et al., 2001), originally developed to evaluate health-related quality of life and healthcare experiences in women with endometriosis. The Italian version of the instrument has been validated by Gioia et al. (2024), and its adaptation in the present study ensured contextual relevance for women with vulvodynia while preserving the conceptual structure of the original tool. A confirmatory factor analysis supported the two-factor solution, showing excellent fit indices (χ^2^/df = 1.92, CFI = 0.97, TLI = 0.96, RMSEA = 0.04 [90% CI = 0.02–0.06], SRMR = 0.03). In our sample, the satisfaction with healthcare professionals scale demonstrated good internal consistency (ω = 0.83), whereas the satisfaction with treatment scale showed acceptable reliability (ω = 0.72).

#### 2.4.2. Dyadic Adjustment Scale

The quality of the couple relationship was assessed using the *Dyadic Adjustment Scale* (DAS) (Spanier, 1976), a widely used instrument for evaluating individuals’ perceptions of their relationships with intimate partners. The scale measures four central dimensions of relational adjustment: dyadic consensus, dyadic satisfaction, dyadic cohesion, and affective expression. It is composed of 32 items rated on Likert-type scales, and it yields both subscale and total scores with higher values indicating greater overall relationship quality. Example items include “Do you and your partner agree about handling family finances?” (dyadic consensus), “How often do you and your partner quarrel?” (dyadic satisfaction), and “Do you confide in your mate?” (dyadic cohesion). The Italian version of the DAS has shown strong psychometric properties (Gentili et al., 2002).

In the present study, both subscale and total scores were considered. Reliability analyses confirmed excellent internal consistency for the total dyadic adjustment score (ω = 0.94). Subscale reliability was also satisfactory, with ω = 0.92 for dyadic consensus, ω = 0.86 for dyadic satisfaction, ω = 0.72 for affective expression, and ω = 0.79 for dyadic cohesion.

#### 2.4.3. Perceived Social Support

Perceived social support was assessed using four items designed to provide a brief global index of informal support relevant to women living with vulvodynia. Participants were asked to indicate their level of agreement with the following statements: “I have good friends who support me,” “I can always count on my family,” “I can rely on colleagues and acquaintances,” and “I can count on a good social support network (e.g., patient associations, peer-support groups, online support communities).” These sources were selected to reflect forms of support frequently described by women with vulvodynia as especially meaningful in managing a stigmatized chronic pain condition. Responses were collected on a 5-point Likert scale ranging from 1 (*strongly disagree*) to 5 (*strongly agree*), with higher scores indicating stronger perceived support. A confirmatory factor analysis supported the unidimensional structure of the scale, yielding satisfactory fit indices (χ^2^/df = 1.84, CFI = 0.98, TLI = 0.96, RMSEA = 0.04 [90% CI = 0.02–0.07], SRMR = 0.03). The scale showed acceptable internal consistency, as reflected by a McDonald’s omega coefficient of 0.73, which supports its reliability in the sample.

#### 2.4.4. General Self-Efficacy Scale

General self-efficacy was measured using the *General Self-Efficacy Scale* (GSE) (Schwarzer & Jerusalem, 1995), an instrument designed to assess individuals’ confidence in their ability to manage a broad range of challenging or unfamiliar situations. The scale is composed of 10 items rated on a 4-point Likert scale, ranging from 1 (*not at all true*) to 4 (*exactly true*). Sample items include: “I can always manage to solve difficult problems if I try hard enough” and “I am confident that I could deal efficiently with unexpected events.” Higher total scores indicate stronger perceived self-efficacy. The Italian version of the GSE has shown robust psychometric properties (Sibilia et al., 1995). In the present study, the scale demonstrated excellent internal consistency, with a McDonald’s omega coefficient of 0.92, further supporting its reliability in this sample.

#### 2.4.5. Perceived Stress Scale

Perceived stress was assessed using the *Perceived Stress Scale* (PSS) (S. Cohen et al., 1983), which is one of the most widely used instruments for evaluating the extent to which individuals perceive situations in their lives as stressful. The scale is composed of 10 items rated on a 5-point Likert scale ranging from 0 (*never*) to 4 (*very often*). Participants were asked to report how frequently they had experienced stress-related thoughts and feelings over the past month. Sample items include “In the last month, how often have you felt that you were unable to control the important things in your life?” and “In the last month, how often have you felt confident about your ability to handle your personal problems?” Higher total scores reflect greater perceived stress. The Italian version of the PSS has shown strong psychometric properties (Mondo et al., 2021). In the present study, the scale demonstrated good internal consistency, with a McDonald’s omega coefficient of 0.86.

#### 2.4.6. General Health Questionnaire-12

The *General Health Questionnaire-12* (GHQ-12) (Goldberg & Williams, 1988) was used to evaluate participants’ psychological well-being, operationalized through indicators of psychological distress and general psychological functioning. This widely used screening tool captures non-specific indicators of psychological functioning and everyday well-being in both community and clinical populations. The GHQ-12 consists of 12 items assessing recent experiences related to concentration, decision-making, sleep, and perceived strain. Each item is rated on a 4-point Likert scale ranging from 0 (*less than usual*) to 3 (*much more than usual*), with higher scores reflecting lower levels of well-being. Example items include “Have you recently been able to concentrate on whatever you are doing?”, “Have you recently lost much sleep over worry?”, and “Have you recently felt capable of making decisions about things?”. The Italian version of the GHQ-12 has demonstrated satisfactory psychometric properties (Piccinelli et al., 1993). In the present study, the scale showed good internal consistency, with a McDonald’s omega coefficient of 0.86.

### 2.5. Statistical Analyses

All statistical analyses were conducted using IBM SPSS Statistics (version 31.0, IBM Corporation, Armonk, NY, USA). Descriptive statistics were computed for all study variables and are reported as means and standard deviations for continuous variables, and as frequencies and percentages for categorical variables. Before conducting the analyses, the distribution of continuous variables was inspected to assess normality. Due to the moderate sample size, skewness and kurtosis values were used instead of formal tests because they provide more reliable indicators of normality in psychological research. Following West et al.’s (1995) guidelines, skewness values exceeding |2| and kurtosis values exceeding |7| were considered evidence of significant deviations from normality.

Confirmatory factor analyses (CFAs) were conducted only for the measures whose dimensionality had not been previously established in Italian samples or for which an adapted or study-specific version was used. Specifically, CFAs were performed for the perceived social support scale and the adapted satisfaction measures. Model fit was assessed using multiple indices, including chi-square to degree of freedom ratio (χ^2^/df), comparative fit index (CFI), Tucker–Lewis index (TLI), root mean square error of approximation (RMSEA), and standardized root mean square residual (SRMR), following conventional cutoffs (Hu & Bentler, 1999). The internal consistency of each measure was evaluated using McDonald’s omega (ω), which provides a more accurate estimate of reliability than Cronbach’s alpha, especially when the assumption of tau-equivalence is unlikely to be met (McDonald, 1999; Sijtsma, 2009). A threshold of ω ≥ 0.70 was adopted to indicate acceptable reliability (Dunn et al., 2014). All scales used in the present study met or exceeded this criterion, as detailed in Section 2.4.

To ensure comparability across measures, all continuous scores were standardized prior to conducting the analyses. Independent-sample t-tests and ANOVAs were used to examine differences between groups based on sociodemographic and illness-related variables. Post hoc pairwise comparisons for ANOVAs were conducted using Tukey’s HSD test. Effect sizes were reported to evaluate the magnitude of these differences. For t tests, effect sizes were reported using Cohen’s d (small = 0.20, medium = 0.50, large = 0.80; J. Cohen, 1988). For ANOVAs, effect sizes were reported using partial eta squared (small = 0.01, medium = 0.06, large = 0.14; J. Cohen, 1988). Then, Pearson correlations were computed to explore associations among the questionnaire scores.

Finally, two hierarchical multiple regression analyses were conducted, using general health and psychological well-being (GHQ-12 score) as the dependent variable. Before conducting the analyses, the assumptions of linearity, homoscedasticity, independence of errors, and normality of residuals were examined. Multicollinearity was evaluated through the variance inflation factor (VIF), with values below 10 generally considered acceptable and values below 5 recommended in the psychological literature as a more conservative criterion (Marcoulides & Raykov, 2018). The first model was estimated on the full sample (N = 533). Sociodemographic variables were entered in the first block, followed by illness-related variables in the second, relational status and social resources in the third, and psychological factors (self-efficacy and perceived stress) in the fourth. A second model was conducted on the subsample of women currently in a romantic relationship (n = 435). In this analysis, the DAS total score was introduced at the third block, replacing the relationship-status variable to directly examine the contribution of relational quality alongside social and psychological resources. The global DAS score was used rather than the individual subscales to avoid potential multicollinearity, as the subdimensions of the measure are highly intercorrelated and would not provide distinct predictive value within a multivariable model. It was not possible to perform a parallel model for single women (n = 98) due to the small sample size, which would have prevented adequate statistical power.

Post hoc sensitivity analyses were performed using G*Power, version 3.1.9.6 (Faul et al., 2009) to determine the minimum detectable effect size (f^2^) given the available sample size, α = 0.05, and power (1-β) = 0.90. Separate analyses were conducted for the relational-social (Block 3) and psychological (Block 4) predictors in the hierarchical regression model, specifying the number of predictors entered at each step and the total number of predictors in the model. A significance threshold of *p* < 0.05 (two-tailed) was adopted for all statistical tests.

## 3. Results

### 3.1. Sensitivity Analysis

The sensitivity analysis indicated that, in the full sample (N = 533), the regression models had 80% power to detect incremental effects as small as f^2^ = 0.02 for both Block 3 and Block 4. In the partnered subsample (n = 435), the models had 80% power to detect incremental effects of at least f^2^ = 0.03 for both Block 3 and Block 4. According to J. Cohen’s (1988) criteria, these values correspond to small effect sizes, suggesting that both models were adequately powered to detect small-to-moderate associations.

### 3.2. Disease-Related Characteristics

In terms of disease duration, almost one third of women reported experiencing vulvodynia symptoms for five to ten years (31.7%), and a further 20.8% had lived with symptoms for more than ten years. Shorter durations were less common, with 22.9% reporting 2–4 years and 14.6% reporting 1–2 years. The time elapsed from symptom onset to receiving a medical diagnosis also varied considerably. Nearly one third of participants (31.5%) reported waiting more than five years for a diagnosis, while smaller proportions received it sooner, including 21.4% within two to four years, 16.7% within six to twelve months, and 15% within the first six months. Pain intensity, classified according to established clinical guidelines, was distributed across mild (0–3), moderate (4–6), and severe (7–10) categories. Within the present sample, 44.1% of women reported severe pain, 29.6% reported moderate pain, and 26.3% reported mild pain.

Regarding symptomatology, the most frequently reported were vulvar pain during intercourse (91.0%), burning sensations (87.8%), vulvar hypersensitivity (80.7%), and vaginal dryness (71.7%). Dyspareunia (67.5%) was also frequently reported. Medical comorbidities were highly prevalent, particularly recurrent cystitis (64.9%) and candidiasis (62.9%). In addition, psychological difficulties were reported by a substantial proportion of participants, with 42.6% indicating anxiety symptoms and 32.8% depressive symptoms.

At the time of the survey, most women (76.7%) were undergoing treatment for vulvodynia. Among these, more than half (53.8%) had started therapy within the previous year, 22% had been in treatment for one to two years, and smaller percentages reported longer treatment durations. The most frequently treatments were physiotherapy (67.9%), pharmacological treatments (65.7%), and psychotherapy (32.6%), often combined within multimodal therapeutic approaches.

### 3.3. Questionnaire Scores

Mean scores and standard deviations for all psychological and relational measures are reported in Table 1. On average, participants reported moderate levels of perceived stress and good levels of general self-efficacy, while scores on the GHQ-12 suggested a notable degree of psychological distress. Satisfaction with healthcare providers and treatments was generally low. Regarding relational adjustment, women currently in a romantic relationship showed moderate dyadic adjustment scores, with variability across the subscales of consensus, satisfaction, cohesion, and affectional expression. Additionally, perceived social support yielded a mean score of 12.51 (SD = 3.74), with possible values ranging from 4 to 20, indicating substantial variability in the extent to which participants felt supported by their social network. All variables displayed acceptable skewness and kurtosis values, supporting the assumption of approximate normality and the use of parametric methods.

### 3.4. Group Comparisons

To further investigate subgroup differences, a series of one-way ANOVAs and independent-samples t-tests were conducted on the standardized scores of psychological, relational, and health-related variables. Age-related analyses revealed significant group differences. General well-being varied significantly across age groups [F(3, 175) = 3.65, *p* = 0.014, η^2^ = 0.02]. Post hoc comparisons showed that women aged 26–35 reported poorer well-being than those aged 36–50 (*p* = 0.023) and those over 50 (*p* = 0.032). No significant differences were found when comparing women of different educational levels across outcomes such as self-efficacy, perceived stress, general well-being, and satisfaction with healthcare and treatments. Analyses by pain intensity revealed significant differences with small-to-moderate effect sizes. Compared with women with mild or moderate pain, women with severe pain reported significantly higher perceived stress [F(2, 530) = 7.84, *p* < 0.001, η^2^ = 0.03)], lower general well-being [F(2, 530) = 9.12, *p* < 0.001, η^2^ = 0.03], and lower satisfaction with treatment [F(2, 530) = 6.47, *p* = 0.002, η^2^ = 0.02]. Significant patterns were also observed for time since diagnosis. Women who waited more than five years for a diagnosis showed higher perceived stress [F(3, 529) = 4.65, *p* = 0.003, η^2^ = 0.03] and lower general well-being [F(3, 529) = 3.92, *p* = 0.009, η^2^ = 0.02]. These effects were small but consistent. Similarly, symptom duration was associated with poorer outcomes. Women who had experienced symptoms for more than ten years reported higher stress and lower satisfaction with care than those with shorter symptom histories [F(3, 529) = 5.41, *p* = 0.0001, η^2^ = 0.03], reflecting small-to-moderate effects. Comparisons by treatment status revealed that women currently undergoing therapy reported significantly higher satisfaction with treatment than those not in therapy [t(531) = −2.72, *p* = 0.007, d = 0.30]. The effect size was small but meaningful. No significant differences emerged for self-efficacy, perceived stress, and general well-being.

Regarding relationship status, there were no significant differences in psychological and health-related variables between those in a romantic relationship and those who were single. However, clear differences emerged when focusing on relationship quality among partnered women. Those with higher dyadic adjustment reported lower perceived stress [t(428) = 4.02, *p* < 0.001, d = 0.41], higher general well-being [t(428) = −4.06, *p* < 0.001, d = 0.39], greater satisfaction with healthcare [t(428) = −3.48, *p* = 0.001, d = 0.35], and higher satisfaction with treatment [t(428) = −4.24, *p* < 0.001, d = 0.41]. These effect sizes were consistently in the small-to-moderate range, highlighting the importance of relationship quality in shaping women’s adaptation.

### 3.5. Correlation Analyses

Pearson’s correlations revealed consistent associations among the study variables (Table 2). Higher satisfaction with healthcare professionals and treatments was positively associated with social support, self-efficacy, and dyadic adjustment. It was also negatively associated with perceived stress and poorer general well-being, as measured by the GHQ-12 (where higher scores indicate lower well-being). Similarly, higher levels of social support and self-efficacy were associated with reduced stress and better well-being, while perceived stress was associated with poorer well-being. As expected, the dimensions of dyadic adjustment were strongly intercorrelated, and higher couple adjustment was consistently associated with more favorable psychosocial outcomes.

### 3.6. Hierarchical Regression Analyses

A hierarchical multiple regression analysis was conducted to identify predictors of general health and psychological well-being (as measured by GHQ-12 scores) in the total sample of women with vulvodynia (N = 533). Model diagnostics confirmed the adequacy of the assumptions with no evidence of multicollinearity (all VIFs < 2). The overall model accounted for 40.6% of the variance in GHQ-12 scores (R^2^ = 0.41, F(21,511) = 16.61, *p* < 0.001). At Step 1, age emerged as a significant predictor, with younger women reporting lower well-being (*β* = −0.13, *p* = 0.005). Educational attainment was also significant: women with a high school diploma reported poorer well-being compared to those with lower educational levels (*β* = 0.21, *p* = 0.031). Relationship status was not a significant predictor. At Step 2, adding illness-related variables significantly improved the model fit (R^2^ = 0.12, *p* < 0.001). Pain intensity was the strongest predictor at this stage, with higher levels of pain being associated with poorer well-being (*β* = 0.27, *p* < 0.001). However, diagnostic delay and symptom duration were not significant predictors once other variables were included. Age remained a significant predictor (*β* = −0.14, *p* = 0.003). At Step 3, relational and social resources accounted for an additional 13.5% of the variance (R^2^ = 0.26, *p* < 0.001). Social support (*β* = −0.17, *p* < 0.001), satisfaction with healthcare professionals (*β* = −0.09, *p* = 0.040), and satisfaction with treatment (*β* = −0.28, *p* < 0.001) were all significant predictors, with higher satisfaction and stronger perceived support associated with better well-being. Pain intensity remained a significant predictor (*β* = 0.14, *p* = 0.002), though its effect size was reduced. Age continued to predict well-being (*β* = −0.14, *p* = 0.001). At Step 4, psychological variables explained an additional 14.7% of the variance (R^2^ = 0.41, *p* < 0.001). In the final model, general self-efficacy emerged as the strongest predictor of well-being (*β* = −0.37, *p* < 0.001), followed by perceived stress (*β* = 0.14, *p* < 0.001). Satisfaction with treatment (*β* = −0.23, *p* < 0.001) and social support (*β* = −0.07, *p* = 0.046) were still significant predictors, while satisfaction with healthcare professionals became marginal (*p* = 0.066). Pain intensity had a smaller, yet significant, effect (*β* = 0.11, *p* = 0.006). Age was still significant (*β* = −0.13, *p* < 0.001), whereas educational attainment was no longer predictive. Table 3 presents the complete results of the regression model.

A parallel analysis was conducted on the subsample of women who were currently in romantic relationships (n = 435). The results were largely consistent with those of the overall sample. Age continued to predict well-being in the first three blocks, though its effect was slightly weaker in the final model (*β* = −0.12, *p* = 0.007). Pain intensity remained significant (*β* = 0.13, *p* = 0.004), but its effect diminished once psychosocial resources were included. Perceived social support (*β* = −0.20, *p* < 0.001) and treatment satisfaction (*β* = −0.23, *p* < 0.001) both emerged as robust predictors of well-being. When DAS was added, its effect was small and not significant (*β* = −0.08, *p* = 0.093), suggesting that dyadic adjustment did not explain additional variance beyond social support and treatment satisfaction. In the final block, psychological factors again emerged as the strongest predictors, with self-efficacy (β = –0.36, *p* < 0.001) and perceived stress (β = 0.15, *p* < 0.001) having the most consistent effects. Table 4 presents the complete results of the regression model.

## 4. Discussion

This study aimed to investigate the psychosocial and developmental factors associated with psychological well-being in Italian women with vulvodynia, adopting a biopsychosocial and lifespan developmental perspective. The findings revealed that well-being is not exclusively determined by medical variables, such as pain intensity or illness duration, but rather emerges from the complex interplay of developmental resources, psychological functioning, and social integration.

Group comparisons offered important preliminary insights. Women experiencing severe pain consistently reported higher stress, poorer well-being, and lower treatment satisfaction than those with mild or moderate pain, underscoring the central role of symptom intensity in influencing daily functioning. Similarly, women with long diagnostic delays and symptom durations exceeding ten years reported lower psychological well-being and higher stress levels. While the cross-sectional design precludes causal interpretation, this pattern aligns with prior literature indicating that prolonged uncertainty and delayed symptom recognition frequently co-occur with an elevated psychological burden in chronic, poorly understood conditions (Skojec et al., 2025). These findings also align with developmental perspectives that conceptualize chronic illness as a non-normative challenge. When symptoms persist for many years and healthcare responses are fragmented, women may experience heightened strain, which can hinder developmental progress and adaptive functioning (Hendry & Kloep, 2002; Mishel, 1990). In contrast, women actively engaged in treatment reported greater satisfaction with care, highlighting that therapeutic involvement may mitigate some of the harmful effects of chronic pain.

Relational and social resources also emerged as highly relevant. While being in a romantic relationship itself did not differentiate psychological outcomes from being single, women who reported greater dyadic adjustment consistently reported higher well-being, lower stress, and greater satisfaction with both healthcare and treatment. These results highlight the developmental significance of intimate partnerships as sources of emotional support and validation, especially during life stages when stability and intimacy are key developmental tasks (Gómez-López et al., 2019; Shanahan, 2000).

The hierarchical regression analyses provided a more comprehensive perspective by simultaneously modeling sociodemographic, illness-related, relational, social, and psychological factors. Age emerged as a significant predictor, as confirmed also by group comparisons. Younger women reported poorer well-being, whereas older participants demonstrated comparatively better adaptation. This finding suggests that vulvodynia may be especially disruptive during emerging and early adulthood. Indeed, during these life stages, vulvodynia can interfere with sexual exploration, the formation of romantic relationships, and the consolidation of educational or occupational pathways (Arnett, 2000; Schulenberg et al., 2004a). Conversely, older women may benefit from greater coping resources accumulated over the life course or from a redefinition of priorities that reduces the salience of pain in everyday functioning. Among illness-related factors, pain intensity was confirmed as the strongest negative predictor of well-being. However, its predictive effect diminished once psychosocial variables were introduced, suggesting that the burden of pain is not deterministic and can be mitigated by contextual and personal resources, consistent with Engel’s (1977) biopsychosocial model. Perceived social support and satisfaction with treatment emerged as significant protective factors, demonstrating the role of external resources in mitigating the impact of chronic symptoms (Thoits, 2011).

The partnered-only analysis provided an additional perspective. When dyadic adjustment was entered into the model, it did not account for unique variance beyond social support and treatment satisfaction, despite its significant bivariate correlations. This pattern suggests that relational quality may influence outcomes indirectly, through the perception of social support and engagement with healthcare rather than through independent pathways. Across both models, psychological resources ultimately emerged as the most robust predictors. Self-efficacy demonstrated the strongest association with well-being, followed by perceived stress. These findings align with Bandura’s (1982) framework, which conceptualizes self-efficacy as a central mechanism of agency that enables individuals to sustain adaptive efforts under adverse conditions. Conversely, high levels of perceived stress appear to amplify vulnerability, reinforcing cycles of increased distress. From a developmental perspective, these results highlight the critical role of agency and self-efficacy when coping with non-normative challenges such as vulvodynia, where stigma, invisibility, and medical uncertainty may disrupt normative developmental trajectories.

The cultural context through which women interpret and manage vulvodynia also deserves careful consideration. Qualitative studies in Italy have shown that vulvodynia is often viewed as a “contested” condition. In this framework, women’s pain is filtered through dominant cultural narratives about femininity, penetrative sexuality, and the legitimacy of women’s suffering (Romaioli & Caprini, 2025). Within these narratives, stigma and a lack of recognition often exacerbate distress, while supportive relationships help women reconstruct meaning and develop adaptive narratives. Our findings are consistent with this perspective, as the strong associations between social support, treatment satisfaction, and psychological well-being suggest that interpersonal validation and positive healthcare experiences can offset the cultural invalidation many women experience.

Evidence from other cultural contexts reinforces the role of sociocultural norms in shaping women’s adjustment. Expectations surrounding femininity, attractiveness, and sexual performance can intensify emotional distress and complicate symptom interpretation (Di Gesto et al., 2025). Studies focusing on women of color reveal additional layers of complexity, showing how racism, stereotypes, and differential validation by clinicians and partners affect the communication and treatment of vulvar pain (Adams et al., 2024). Similarly, partner-focused studies highlight the impact of dominant cultural scripts of masculinity, which oscillate between ideals of virility and expectations of emotional restraint. These scripts can either alleviate or intensify the burden associated with vulvodynia (Myrtveit-Stensrud et al., 2025; Myrtveit-Stensrud et al., 2023; Myrtveit-Stensrud et al., 2024).

Beyond relational and cultural processes, sociological and anthropological perspectives contextualize women’s experiences of vulvodynia within broader structural frameworks. Sociological research emphasizes how gender inequalities affect access to care, the credibility of women’s health concerns, and the emotional labor necessary for being perceived as a “credible” patient (Hoffmann & Tarzian, 2001; Werner & Malterud, 2003). Anthropological studies of embodied suffering demonstrate that pain is interpreted through culturally patterned narratives, moral expectations, and power relations within medical encounters (Good, 1994; Kleinman, 1988; Scheper-Hughes & Lock, 2009). These insights resonate with our findings that variables such as social support and treatment satisfaction function as interpersonal resources and buffers within structural and cultural environments that may otherwise delegitimize women’s pain.

Integrating these disciplinary perspectives highlights the importance of situating vulvodynia within its broader sociocultural and institutional contexts and underscores the need for interdisciplinary approaches capable of capturing the full complexity of women’s lived experiences. Together, these contributions—including the present study—emphasize the necessity of cross-cultural research examining how sociocultural norms and gendered expectations intersect with personal and relational resources to influence women’s adaptation to vulvodynia.

Overall, this study demonstrates that the well-being of women with vulvodynia reflects the convergence of medical, social, and psychological processes within a developmental trajectory. Pain and illness characteristics impose significant constraints, but it is the availability of adequate social support, the quality of healthcare experiences, and, above all the presence of psychological resources that ultimately determine women’s adaptation. These findings not only confirm the value of integrated biopsychosocial approaches (Engel, 1977), but also extend them by highlighting how adaptation is embedded within the developmental tasks and transitions of adulthood.

### 4.1. Implications for Emotional Support Interventions in Vulvodynia

The present findings have direct implications for designing interventions that provide emotional support to women with chronic conditions, such as vulvodynia. Specifically, the strong predictive role of self-efficacy and perceived stress suggests that psychological programs should focus on strengthening agency and coping strategies. This can be achieved through cognitive-behavioral techniques, mindfulness-based interventions, or resilience training, for example. Similarly, the protective role of social support underscores the importance of group-based approaches and peer support programs, which can reduce stigma, promote shared experiences, and enhance a sense of belonging. Finally, the link between satisfaction with treatment and well-being suggests that interventions designed to improve communication between patients and healthcare providers could significantly improve women’s adaptation to their condition and reduce its emotional burden.

### 4.2. Role of Healthcare Professionals

Healthcare professionals play a critical role in addressing the psychological impact of vulvodynia. In addition to managing pain, clinicians can provide emotional support by validating women’s experiences, acknowledging the stigma surrounding the condition, and offering compassionate, patient-centered care. Training gynecologists, physiotherapists, and psychologists to adopt an integrated, empathetic approach could improve treatment satisfaction and enhance psychological well-being indirectly. Furthermore, interdisciplinary care pathways combining medical, psychological, and relational support represent a promising direction for clinical practice and policy development.

### 4.3. Limitations and Future Directions

Several limitations should be considered when interpreting these findings. First, the cross-sectional design precludes causal inference. Longitudinal data are needed to clarify how developmental trajectories unfold through the interplay of illness-related challenges and psychosocial resources over time. Second, reliance on self-reporting could have introduced recall or reporting biases, especially regarding retrospective information on symptom onset and diagnosis. Third, the voluntary online recruitment strategy may have introduced self-selection bias. Since participation occurred through patient associations, online communities, and social media channels, the sample may overrepresent women who are more digitally engaged or connected to support networks, thus limiting the generalizability of the findings. Additionally, since the study did not systematically collect information on socioeconomic status or ethnicity, we could not evaluate diversity in these areas. Fourth, although the study included a wide range of psychosocial and illness-related variables, additional factors, such as personality traits, coping strategies, and cultural perceptions of femininity, were not assessed and warrant further investigation. Finally, the global perceived social support index was developed specifically for this study. While it demonstrated good psychometric properties and captured support forms particularly salient for women with vulvodynia, incorporating validated global measures, such as the F-SozU (Kliem et al., 2015), in future research would enhance comparability across studies.

Therefore, future work should adopt longitudinal and mixed-methods designs to capture the dynamic and subjective aspects of vulvodynia adaptation. Structural equation modeling (SEM) could be used analytically to test theory-driven pathways among pain, psychosocial resources, and psychological well-being, including indirect effects, such as social support or self-efficacy mediating the association between pain intensity and well-being. It could also include moderation by clinically meaningful factors, such as pain severity, treatment engagement, age, or relationship status. In multi-wave studies, cross-lagged panel or latent growth models can clarify temporal ordering and quantify reciprocal influences, such as those between stress and pain. Strengthening external validity requires increasing sample diversity and oversampling underrepresented groups. Integrating quantitative data with qualitative accounts could offer a more comprehensive understanding of how women mobilize resources, sustain resilience, and navigate developmental challenges in the context of chronic pain.

Future studies should also examine the influence of sociocultural narratives surrounding femininity, stigma, and partner expectations. Research suggests that cultural scripts about women’s bodies, sexuality, and relational roles shape the interpretation and legitimacy of vulvar pain, with consequences for psychological adjustment and help-seeking. Cross-cultural and diversity-focused studies would help clarify how sociocultural and structural factors amplify or mitigate the burden of vulvodynia across different populations. Future research would also benefit from interdisciplinary approaches integrating sociological and anthropological frameworks to examine how structural inequalities, cultural narratives of femininity, and embodied experiences of pain shape women’s adaptation to vulvodynia.

## 5. Conclusions

This study provides new evidence showing that the psychological well-being for women with vulvodynia is influenced by medical, developmental, psychological, and social factors. Adopting a lifespan perspective reveals how the onset and persistence of chronic vulvar pain can disrupt typical activities at various stages of adulthood, especially for younger women engaged in identity exploration, relationship formation, and career development. Situating vulvodynia within this developmental framework emphasizes the importance of recognizing it as a medical and psychosocial challenge that unfolds throughout life.

Future research should build on these insights by using longitudinal designs to explore the dynamic interplay between illness-related challenges and psychosocial resources over time. Researchers should pay special attention to contextual factors, such as stigma, cultural narratives of femininity, and partner dynamics, which may amplify or mitigate the condition’s burden. Examining how these factors operate at different stages of adulthood will advance our understanding of women’s adaptation and resilience. Ultimately, integrating a developmental lens into research and practice can deepen theoretical knowledge and inform interventions responsive to the diverse needs of women living with chronic conditions throughout their lives.

## Figures and Tables

**Table 1:** Descriptive Statistics for Study Variables.

	N	M	SD	Skewness	Kurtosis
Satisfaction with Healthcare Providers	533	8.00	4.32	0.37	−0.81
Satisfaction with Treatment	533	4.84	2.57	0.46	0.02
Perceived Social Support	533	12.51	3.74	−0.10	−0.68
GSE	533	26.73	6.37	−0.01	−0.39
PSS	533	17.50	3.71	−0.26	0.97
GHQ-12	533	25.38	7.40	−0.25	−0.26
DAS—Total Score	423	107.17	24.29	−0.78	0.29
DAS—Dyadic Consensus	423	46.58	12.99	−1.09	1.09
DAS—Dyadic Satisfaction	423	36.78	8.08	−0.91	0.48
DAS—Affectional Expression	423	7.53	2.98	−0.28	−0.77
DAS—Dyadic Cohesion	423	16.29	4.65	−0.67	0.03

Note. GSE = General Self-Efficacy Scale; PSS = Perceived Stress Scale; GHQ-12 = General Health Questionnaire-12; DAS = Dyadic Adjustment Scale. DAS scores and subscales were computed only for participants currently in a romantic relationship (N = 423).

**Table 2:** Correlations for Study Variables.

	1		2		3		4		5		6		7		8		9		10		11		12	
1. Age	—																							
2. Satisfaction with Healthcare Providers	0.043		—																					
3. Satisfaction with Treatment	−0.019		0.377	***	—																			
4. Perceived Social Support	−0.051		0.225	***	0.209	***	—																	
5. GSE	0.045		0.125	**	0.173	***	0.244	***	—															
6. PSS	−0.000		−0.142	**	−0.195	***	−0.180	***	−0.175	***	—													
7. GHQ-12	−0.139	**	−0.270	***	−0.390	***	−0.262	***	−0.484	***	0.283	***	—											
8. DAS—Dyadic Consensus	−0.181	***	0.158	**	0.159	**	0.110	*	0.006		−0.127	**	−0.161	***	—									
9. DAS—Dyadic Satisfaction	−0.207	***	0.224	***	0.205	***	0.178	***	0.091		−0.138	**	−0.229	***	0.659	***	—							
10. DAS—Affectional Expression	−0.162	***	0.143	**	0.241	***	0.125	*	0.017		−0.103	*	−0.141	**	0.622	***	0.593	***	—					
11. DAS—Dyadic Cohesion	−0.117	*	0.175	***	0.130	**	0.274	***	0.191	***	−0.109	*	−0.230	***	0.463	***	0.582	***	0.378	***	—			
12. DAS—Total Score	−0.208	***	0.210	***	0.207	***	0.186	***	0.072		−0.148	**	−0.223	***	0.919	***	0.870	***	0.725	***	0.679	***	—	

Note. GSE = General Self-Efficacy Scale; PSS = Perceived Stress Scale; GHQ-12 = General Health Questionnaire-12; DAS = Dyadic Adjustment Scale. All scores were standardized (z-scores) prior to analysis. DAS scores and subscales were computed only for participants currently in a romantic relationship (N = 423). * *p* < 0.05, ** *p* < 0.01, *** *p* < 0.001.

**Table 3:** Hierarchical Regression Results for General Well-Being in the Full Sample (N = 533).

	Model 1		Model 2		Model 3		Model 4	
	*B*	*β*	*B*	*β*	*B*	*β*	*B*	*β*
Age	−0.01	−0.13 **	−0.01	−0.14 **	−0.01	−0.14 **	−0.01	−0.13 ***
*Education*								
Middle school	Ref.		Ref.		Ref.		Ref.	
High school diploma	0.44	0.21 *	0.43	0.21	0.24	0.12	0.001	0.001
Bachelor’s degree	0.14	0.06	0.14	0.06	−0.02	−0.008	−0.18	−0.08
Master’s degree	0.15	0.07	0.22	0.09	0.13	0.06	−0.02	−0.01
Postgraduate specialization	0.007	0.002	0.10	0.03	−0.004	−0.001	−0.08	−0.02
*Romantic relationship*								
No	Ref.		Ref.		Ref.		Ref.	
Yes	−0.09	−0.03	−0.03	−0.01	0.051	0.020	−0.02	−0.006
*Symptom duration*								
<1 year			Ref.		Ref.		Ref.	
1–2 years			−0.12	−0.04	−0.11	−0.04	−0.13	−0.04
2–4 years			0.01	0.004	−0.03	−0.01	−0.09	−0.04
5–10 years			0.002	0.001	−0.08	−0.04	−0.07	−0.03
>10 years			−0.07	−0.03	−0.16	−0.07	−0.18	−0.07
*Time since diagnosis*								
<6 months			Ref.		Ref.		Ref.	
6–12 months			0.15	0.06	0.09	0.03	0.14	0.05
1–2 years			−0.10	−0.04	−0.21	−0.08	−0.11	−0.04
2–4 years			−0.04	−0.02	−0.06	−0.02	0.02	0.01
>5 years			−0.02	−0.008	−0.08	−0.04	−0.04	−0.02
Current pain intensity			0.13	0.26 ***	0.07	0.13 **	0.05	0.11 **
*Currently in treatment*								
No			Ref.		Ref.		Ref.	
Yes			−0.04	−0.01	0.000	0.000	−0.03	−0.01
Perceived social support					−0.17	−0.17 ***	−0.07	−0.07 *
Satisfaction with healthcare professionals					−0.09	−0.09 *	−0.07	−0.07
Satisfaction with treatment					−0.28	−0.28 ***	−0.23	−0.23 ***
GSE							−0.37	−0.37 ***
PSS							0.14	0.14 ***
R^2^	0.05		0.12		0.26		0.41	
ΔR^2^	-		0.07		0.13		0.15	

Note. N = 533. GSE = General Self-Efficacy Scale; PSS = Perceived Stress Scale. * *p* < 0.05. ** *p* < 0.01. *** *p* < 0.001.

**Table 4:** Hierarchical Regression Results for General Well-Being in Women with Romantic Relationships (n = 435).

	Model 1		Model 2		Model 3		Model 4	
	*B*	*β*	*B*	*β*	*B*	*β*	*B*	*β*
Age	−0.01	−0.15 **	−0.01	−0.14 **	−0.02	−0.18 ***	−0.01	−0.17 ***
*Education*								
Middle school	Ref.		Ref.		Ref.		Ref.	
High school diploma	0.31	0.15	0.35	0.17	0.14	0.07	−0.06	−0.03
Bachelor’s degree	0.04	0.02	0.06	0.02	−0.10	−0.04	−0.27	−0.11
Master’s degree	0.05	0.02	0.13	0.05	0.03	0.01	−0.11	−0.05
Postgraduate specialization	−0.06	−0.02	0.11	0.03	0.01	0.004	−0.05	−0.01
*Symptom duration*								
<1 year			Ref.		Ref.		Ref.	
1–2 years			−0.07	−0.02	−0.10	−0.03	−0.10	−0.03
2–4 years			0.11	0.04	0.09	0.04	−0.003	−0.001
5–10 years			0.14	0.06	0.08	0.04	0.05	0.02
>10 years			0.05	0.02	0.001	0.001	−0.10	−0.04
*Time since diagnosis*								
<6 months			Ref.		Ref.		Ref.	
6–12 months			−0.11	−0.04	−0.06	−0.02	−0.02	−0.009
1–2 years			−0.26	−0.09	−0.35	−0.13	−0.24	−0.08
2–4 years			−0.30	−0.12	−0.31	−0.13	−0.13	−0.05
>5 years			−0.22	−0.11	−0.31	−0.15	−0.20	−0.09
Current pain intensity			0.14	0.27 ***	0.07	0.14 **	0.06	0.12 **
*Currently in treatment*								
No			Ref.		Ref.		Ref.	
Yes			0.03	0.01	0.09	0.04	0.06	0.02
Perceived social support					−0.25	−0.24 ***	−0.13	−0.12 *
Satisfaction with healthcare professionals					−0.05	−0.05	−0.03	−0.04
Satisfaction with treatment					−0.26	−0.26 ***	−0.21	−0.21 ***
DAS					−0.06	−0.06	−0.08	−0.08
GSE							−0.38	−0.37 ***
PSS							0.16	0.16 ***
R^2^	0.04		0.12		0.29		0.44	
ΔR^2^			0.07		0.17		0.16	

Note. DAS = Dyadic Adjustment Scale; GSE = General Self-Efficacy Scale; PSS = Perceived Stress Scale. * *p* < 0.05. ** *p* < 0.01. *** *p* < 0.001.

## Data Availability

The data presented in this study are available on request from the corresponding author due to privacy restrictions.
